# Expression of Chemerin and Its Receptors in the Porcine Hypothalamus and Plasma Chemerin Levels during the Oestrous Cycle and Early Pregnancy

**DOI:** 10.3390/ijms20163887

**Published:** 2019-08-09

**Authors:** Nina Smolinska, Marta Kiezun, Kamil Dobrzyn, Edyta Rytelewska, Katarzyna Kisielewska, Marlena Gudelska, Ewa Zaobidna, Krystyna Bogus-Nowakowska, Joanna Wyrebek, Kinga Bors, Grzegorz Kopij, Barbara Kaminska, Tadeusz Kaminski

**Affiliations:** Department of Animal Anatomy and Physiology, Faculty of Biology and Biotechnology, University of Warmia and Mazury in Olsztyn, Oczapowskiego 1A, 10-719 Olsztyn-Kortowo, Poland; nina.smolinska@uwm.edu.pl (N.S.); marta.kiezun@uwm.edu.pl (M.K.); kamil.dobrzyn@uwm.edu.pl (K.D.); edyta.rytelewska@uwm.edu.pl (E.R.); katarzyna.kisielewska@uwm.edu.pl (K.K.); marlena.gudelska@uwm.edu.pl (M.G.); ewa.zaobidna@uwm.edu.pl (E.Z.); boguska@uwm.edu.pl (K.B.-N.); joanna.wyrebek@uwm.edu.pl (J.W.); kinga.bors@uwm.edu.pl (K.B.); grzegorzkopij@gmail.com (G.K.); barbara.kaminska@uwm.edu.pl (B.K.)

**Keywords:** chemerin, chemerin receptors, hypothalamus, oestrous cycle, early pregnancy, pig

## Abstract

Chemerin (CHEM) may act as an important link integrating energy homeostasis and reproductive functions of females, and its actions are mediated by three receptors: chemokine-like receptor 1 (CMKLR1), G protein-coupled receptor 1 (GPR1), and C-C motif chemokine receptor-like 2 (CCRL2). The aim of the current study was to compare the expression of CHEM and its receptor (CHEM system) mRNAs (quantitative real-time PCR) and proteins (Western blotting and fluorescent immunohistochemistry) in the selected areas of the porcine hypothalamus responsible for gonadotropin-releasing hormone production and secretion: the mediobasal hypothalamus, preoptic area and stalk median eminence during the oestrous cycle and early pregnancy. Moreover, plasma CHEM concentrations were determined using ELISA. The expression of CHEM system has been demonstrated in the porcine hypothalamus throughout the luteal phase and follicular phase of the oestrous cycle, and during early pregnancy from days 10 to 28. Plasma CHEM levels and concentrations of transcripts and proteins of CHEM system components in the hypothalamus fluctuated throughout pregnancy and the oestrous cycle. Our study was the first experiment to demonstrate the presence of CHEM system mRNAs and proteins in the porcine hypothalamus and the correlations between the expression levels and physiological hormonal milieu related to the oestrous cycle and early pregnancy.

## 1. Introduction

Chemerin (CHEM), encoded by the gene retinoic acid receptor responder 2 (*RARRES2*) or tazarotene-induced gene 2 (*TIG2*), is a chemotactic factor for immune cells engaged in the processes of innate and acquired immunity [1]. CHEM is also an adipokine protein produced and secreted by the adipose tissue, which is considered to be involved in the major metabolic and inflammatory processes [2,3]. In mammalian cells, CHEM is initially synthesised as a 163 amino acid (aa) pre-prochemerin. The N-terminal truncation of 20 aa signal peptide results in the release of 143 residue prochemerin into the blood. Several isoforms of CHEM have been reported that are dependent on the proteolytic cleavage at its C-terminus by various serine and cysteine proteases. Thus, this process serves as a key regulatory mechanism to determine the local and systemic concentrations of active CHEM which exerts local biological actions [4]. In the plasma from healthy humans, inert CHEM precursor is the dominant isoform. However, the different CHEM isoforms in human blood (CHEM-A155, -S157 and -K158), cerebrospinal fluid (CHEM-K158), hemofiltrate (CHEM-F154) and synovial fluid (CHEM-K158) have also been reported, thus indicating that complex prochemerin processing occurs in vivo [5].

CHEM binds with three G-protein coupled receptors: chemokine-like receptor 1 (CMKLR1), in humans also termed as chemerin receptor 23 (ChemR23), G protein-coupled receptor 1 (GPR1), and C-C motif chemokine receptor-like 2 (CCRL2). Of these, CMKLR1 has been the best investigated, and it uses ERK1/2 and Akt kinases for signal transduction. GPR1 is structurally similar to CMKLR1 but its biological function has not been fully investigated. Nevertheless, GPR1 probably has different functions from CMKLR1, because of its presence in different tissues. CCRL2 is clearly different from the two other receptors. It is unable to transduce signals, but binds the N-terminal region of CHEM, exposing the C-terminal region of the hormone molecule to CMKLR1 receptors on the adjacent cells. Therefore, its role is limited to presenting the adipokine to the neighbouring cells with CMKLR1 receptors. The various CHEM isoforms are implied to regulate a biochemical cascade by competition binding to the corresponding receptors. Of these, CHEM-S157 and CHEM-F156 demonstrate the highest affinity to CMKLR1, while other isoforms such as prochemerin, CHEM-K158, or CHEM-F154 bind to the receptor with lower affinity (for review see [6]).

Of all four components of the CHEM system (the hormone and its three receptors), most studies have been focused on CHEM alone and CMKLR1. The adipokine mRNA expression was found mainly in the mouse liver, white adipose tissue and placenta, with lower levels also in the ovaries [2], and in the human liver and pancreas [7]. The expression of the *RARRES2* gene was also found in the ovaries of women [8] and rodents [2,9], and in the hypothalamus of mice and rats [10,11]. CMKLR1 gene expression in mice was detected mainly in the white adipose tissue, with lower levels found in the lungs, heart and placenta [2]. In humans, the highest expression of this receptor was identified in the lymph nodes and spleen [8]. The expression of CMKLR1 was identified in the ovaries of women [8] and rats [9], and in the hypothalamus of rats [11,12]. GPR1 is predominantly expressed in the central nervous system of humans [13] although the expression was also found in the mouse adipose tissue, human skin [14] and granulosa cells [8]. The third CHEM receptor, CCRL2, was also found in the hypothalamus of rats [11]. Most of the research focused on the expression of the CHEM system has been carried out on humans and rodents and data related to farm animals, including pigs, are still missing.

There is a close relationship between nutritional status and reproductive success in animals. The metabolic processes and reproductive system functions are controlled by a number of hormones. It can be assumed that in addition to hormones affecting only the selected metabolic processes or reproductive organs or structures, there are also other hormones creating a link controlling both the metabolic status and reproductive system functions. Based on sparse literature data, a hypothesis can be put forward that CHEM is one such hormone. CHEM is likely to have pleiotropic properties. It influences, for example, food intake, energy homeostasis, adipose tissue function, obesity-related parameters (BMI, blood pressure, cholesterol level), and modulates insulin sensitivity [6,15]. Reduced feed intake, body mass and adiposity, and higher glucose tolerance was found in mice with a disrupted *CMKLR1* gene as compared to the control animals [16]. Moreover, the adipokine is closely related to the female reproductive process. It was described as an important regulator of ovarian steroidogenesis and follicular development [8,9,17,18]. High circulating CHEM levels appear to be associated with pregnancy disorders such as polycystic ovary syndrome (PCOS) [19], pre-eclampsia [20] and endometriosis [21]. It has been also reported that CHEM production in the uterus is up-regulated during decidualization, and that CHEM could play a crucial role in vascular remodelling during early pregnancy of women [22].

In the existing body of research, there are no studies investigating the expression of the CHEM system in the hypothalamic structures responsible for the synthesis of gonadotropin-releasing hormone (GnRH) and the possible impact of hormonal status of the animals connected with the phase of the oestrous cycle and early pregnancy on CHEM and its receptors expression. For this reason, the aim of the present study was to investigate the expression of CHEM system genes and proteins in the specialised hypothalamic structures (mediobasal hypothalamus—MBH, preoptic area—POA, stalk median eminence—SME) involved in the synthesis and secretion of GnRH (the key hypothalamic factor controlling the pituitary gland and, indirectly, ovaries), and serum CHEM levels during the oestrous cycle and early pregnancy, associated with the implantation of embryos.

## 2. Results

### 2.1. The Distribution of CHEM, CMKLR1, GPR1 and CCRL2 in the Porcine Hypothalamus

**Figure 1:**
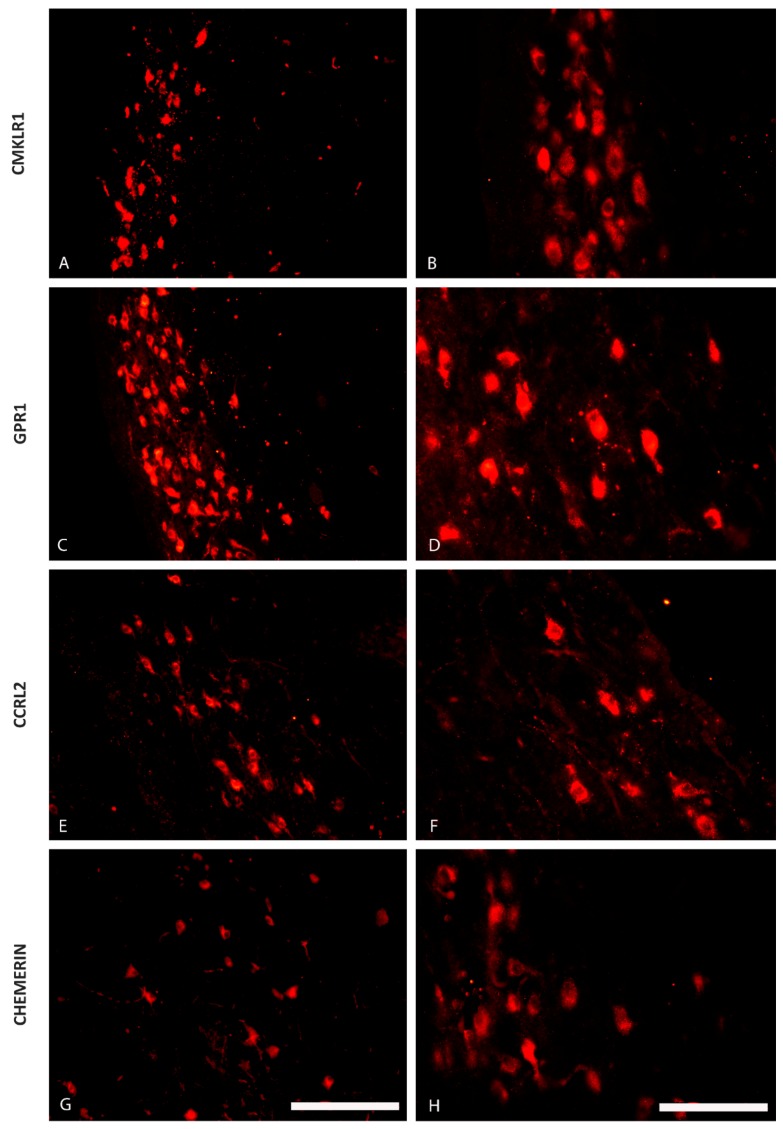
The distribution of the chemerin system in the porcine mediobasal hypothalamus (MBH) during the oestrous cycle. Immunoreactivity of chemokine-like receptor 1 (CMKLR1; **A** and **B**), G protein-coupled receptor 1 (GPR1; **C** and **D**), C-C motif chemokine receptor-like 2 (CCRL2; **E** and **F**) and chemerin (**G** and **H**) during the oestrous cycle (days 10 to 12) in the hypothalamus of the pig at the level of the paraventricular nucleus. Scale bar: 200 µm, applies to A, C, E, G; scale bar: 100 µm, applies to B, D, F, H.

**Figure 2:**
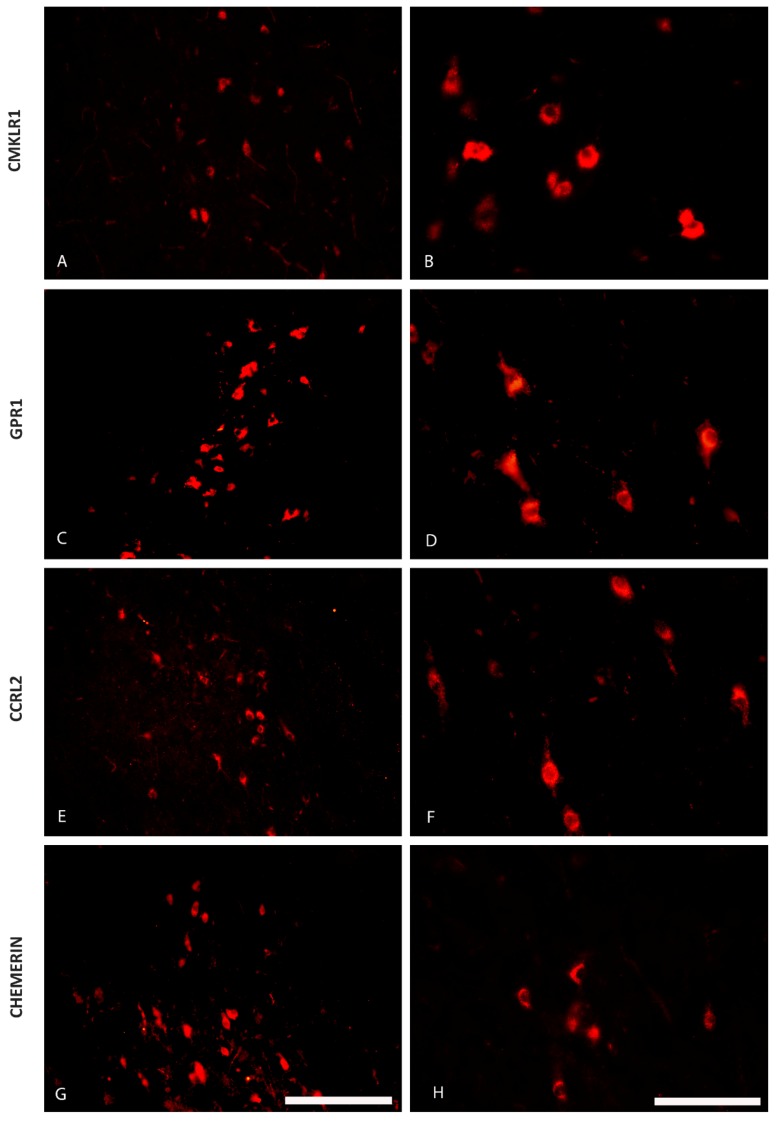
The distribution of the chemerin system in the porcine preoptic area (POA) during the oestrous cycle. Immunoreactivity of chemokine-like receptor 1 (CMKLR1; **A** and **B**), G protein-coupled receptor 1 (GPR1; **C** and **D**), C-C motif chemokine receptor-like 2 (CCRL2; **E** and **F**) and chemerin (**G** and **H**) during the oestrous cycle (days 10 to 12) in the hypothalamus of the pig at the level of the preoptic area. Scale bar: 200 µm, applies to A, C, E, G; scale bar: 100 µm, applies to B, D, F, H.

**Figure 3:**
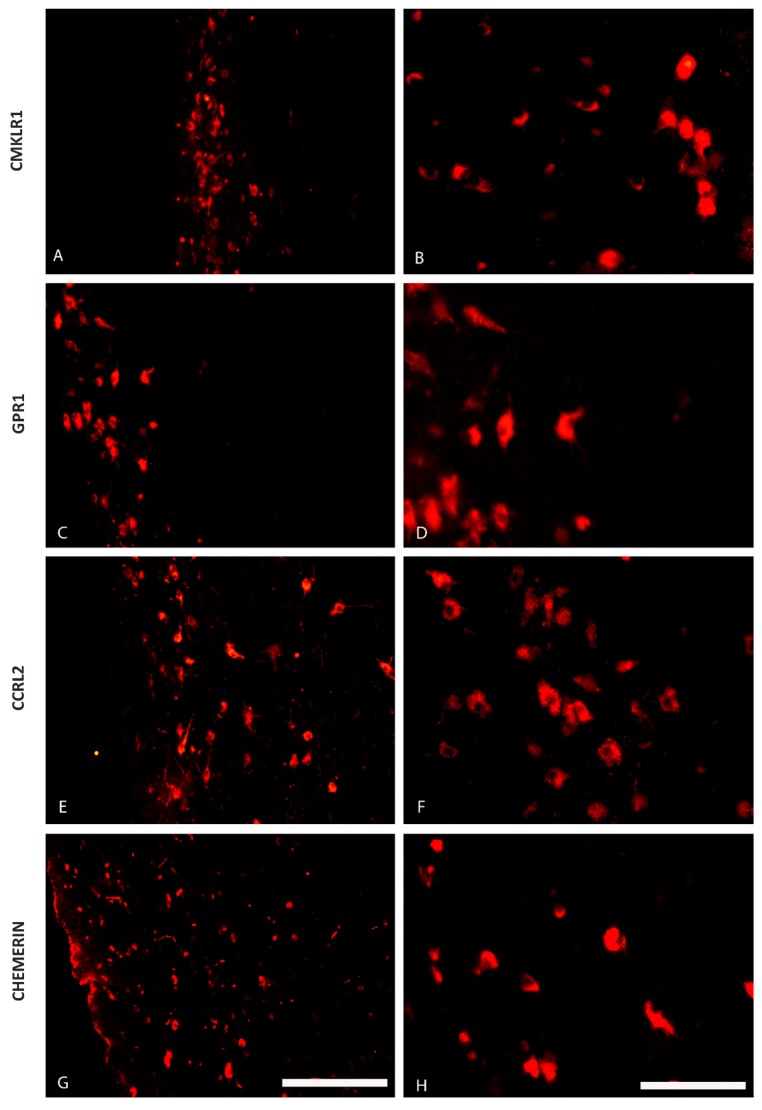
The distribution of the chemerin system in the porcine mediobasal hypothalamus (MBH) during early pregnancy. Immunoreactivity of chemokine-like receptor 1 (CMKLR1; **A** and **B**), G protein-coupled receptor 1 (GPR1; **C** and **D**), C-C motif chemokine receptor-like 2 (CCRL2; **E** and **F**) and chemerin (**G** and **H**) during early pregnancy (days 15 to 16) in the hypothalamus of the pig at the level of the paraventricular nucleus. Scale bar: 200 µm, applies to A, C, E, G; scale bar: 100 µm, applies to B, D, F, H.

**Figure 4:**
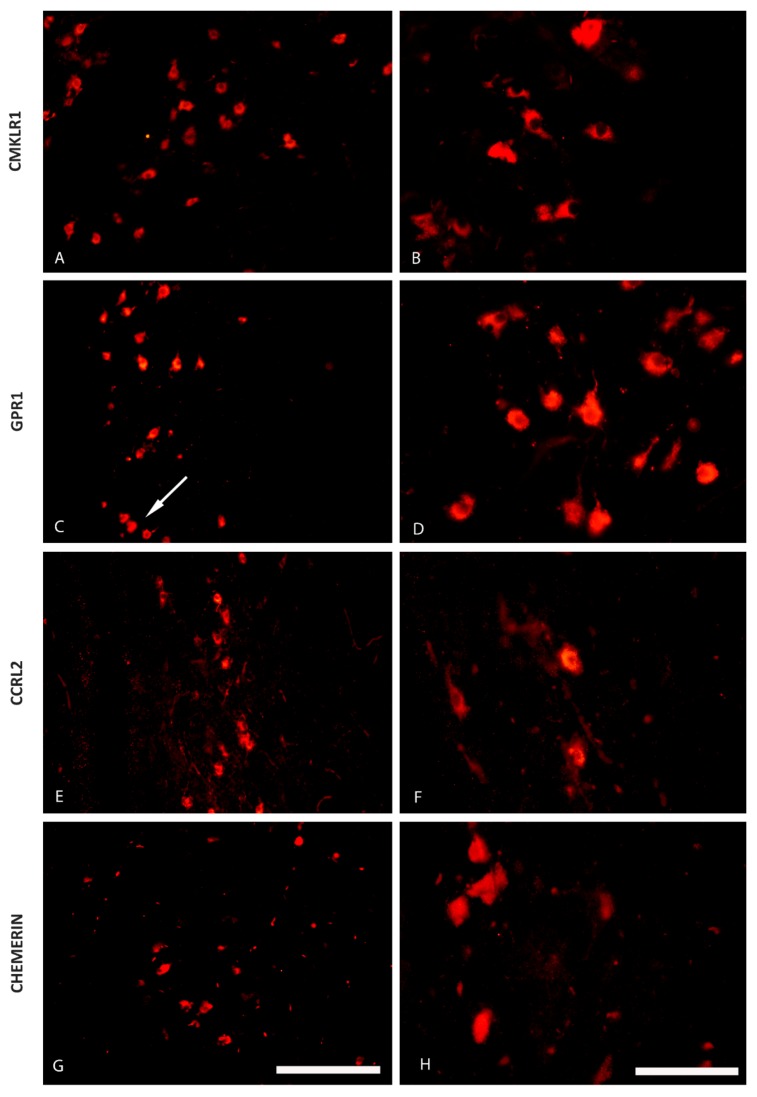
The distribution of the chemerin system in the porcine preoptic area (POA) during early pregnancy. Immunoreactivity of chemokine-like receptor 1 (CMKLR1; **A** and **B**), G protein-coupled receptor 1 (GPR1; **C** and **D**), C-C motif chemokine receptor-like 2 (CCRL2; **E** and **F**) and chemerin (**G** and **H**) during early pregnancy (days 15 to 16) in the hypothalamus of the pig at the level of the preoptic area. The white arrow indicates the diagonal band of Broca. Scale bar: 200 µm, applies to A, C, E, G; scale bar: 100 µm, applies to B, D, F, H.

The immunofluorescence staining has shown the presence of CHEM and its 3 receptors—CMKLR1, GPR1 and CCRL2—in some regions of the pig hypothalamus both during the oestrous cycle (days 10 to 12; Figure 1 and Figure 2) as well as during early gestation (days 15 to 16 of pregnancy; Figure 3 and Figure 4). CHEM-immunoreactive (CHEM-IR) (Figure 1G,H and Figure 3G,H), GPR1-immunoreactive (GPR1-IR) (Figure 1C,D and Figure 3C,D) and CCRL2-immunoreactive (CCRL2-IR) (Figure 1E,F and Figure 3E,F) neurons were the most abundant in the paraventricular nucleus, which is the part of MBH, both during early gestation and during the oestrous cycle. However, CMKLR1-immunoreactive (CMKLR1-IR) cells in this brain region displayed slightly weaker immunoreactivity (Figure 1A,B and Figure 3A,B). Other hypothalamic regions which showed immunoreactivity of CHEM and its receptors were the preoptic region (Figure 2 and Figure 4) and the anterior hypothalamic area. POA exhibited high immunoreactivity of CHEM (Figure 2G,H and Figure 4G,H), GPR1 (Figure 2C,D and Figure 4C,D), CCRL2 (Figure 2E,F and Figure 4E,F) and CMKLR1 (Figure 2A,B and Figure 4A,B). In the anterior hypothalamic area, only single CHEM-IR and CMKLR1-IR cells were identified. Additionally, in the diagonal band of Broca, a part of POA, GPR1 showed very high immunoreactivity (Figure 4C), whereas CHEM-IR and CMKLR1-IR cells were observed sporadically.

### 2.2. CHEM System Gene and Protein Expression in MBH during the Oestrous Cycle

**Figure 5:**
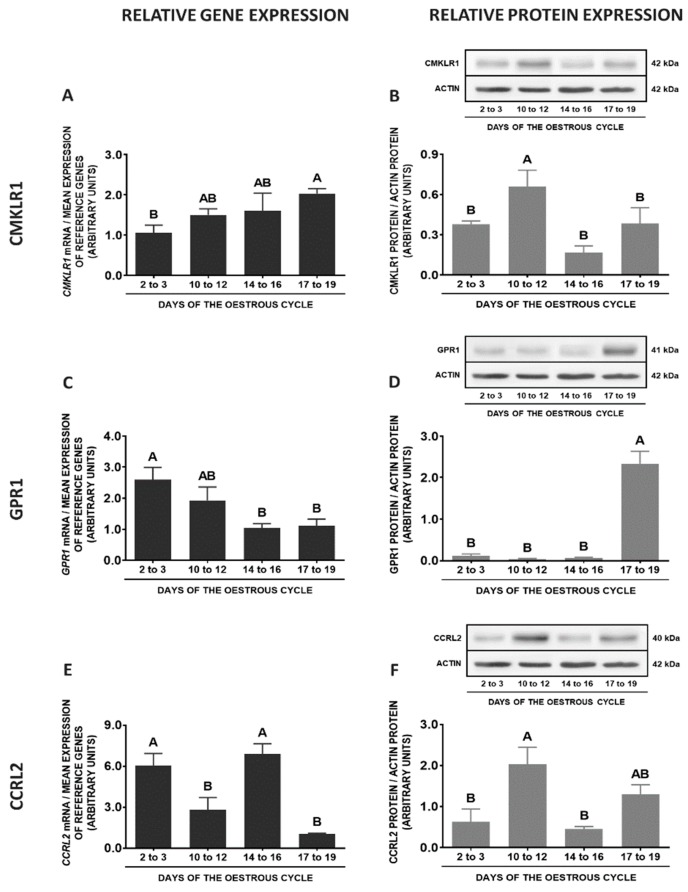

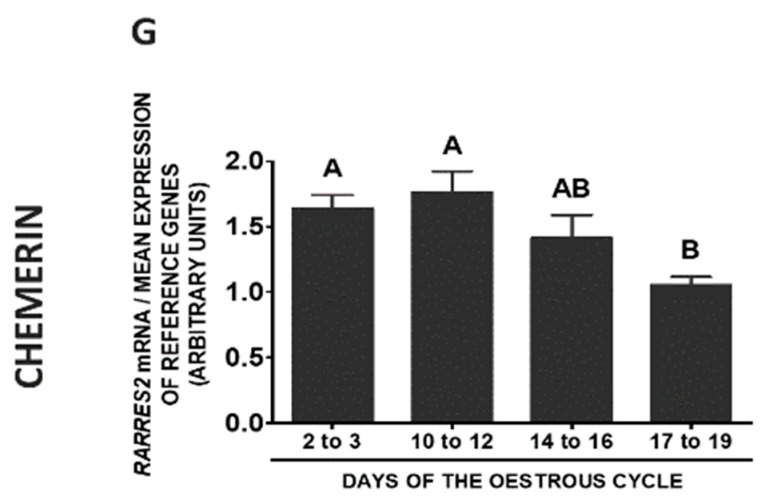
Chemerin system expression in the porcine mediobasal hypothalamus (MBH) during the oestrous cycle. Gene and protein expression of chemokine-like receptor 1 (CMKLR1; **A** and **B**), G protein-coupled receptor 1 (GPR1; **C** and **D**) and C-C motif chemokine receptor-like 2 (CCRL2; **E** and **F**), as well as chemerin (*RARRES2*) mRNA content (**G**) in the porcine MBH on days: 2 to 3, 10 to 12, 14 to 16 and 17 to 19 of the oestrous cycle. Gene expression was determined by qPCR. Protein expression was determined by Western blotting; upper panels: representative immunoblots; lower panels: densitometric analysis of CMKLR1/GPR1/CCRL2 proteins relative to actin protein. Representative actin blots are identical for all chemerin receptors though proteins have been analysed on different gels. Results are presented as means ± SEM (*n* = 5). Bars with different superscripts differ (one-way ANOVA at *p* < 0.05 followed by Tuckey *post-hoc* test at *p* < 0.05).

During the oestrous cycle, the concentrations of *CMKLR1* mRNA were significantly higher on days 17 to 19 when compared to days 2 to 3, whereas protein contents of this receptor were higher on days 10 to 12 of the cycle than in other studied periods of the cycle (Figure 5A,B; *p* < 0.05). *GPR1* gene expression during the cycle was the highest on days 2 to 3 in relation to days 14 to 16 and 17 to 19. The protein contents of GPR1 were the highest on days 17 to 19 when compared to other studied periods of the cycle (*p* < 0.05; Figure 5C,D). Higher *CCRL2* mRNA expression was noted on days 2 to 3 and 14 to 16, whereas protein contents of this receptor were greater on days 10 to 12 in comparison to days 2 to 3 and 14 to 16 (*p* < 0.05; Figure 5E,F). The highest expression of *RARRES2* was observed on days 2 to 3 and 10 to 12 when compared to days 17 to 19 of the cycle (*p* < 0.05; Figure 5G).

### 2.3. CHEM System Gene and Protein Expression in MBH during Pregnancy

**Figure 6:**
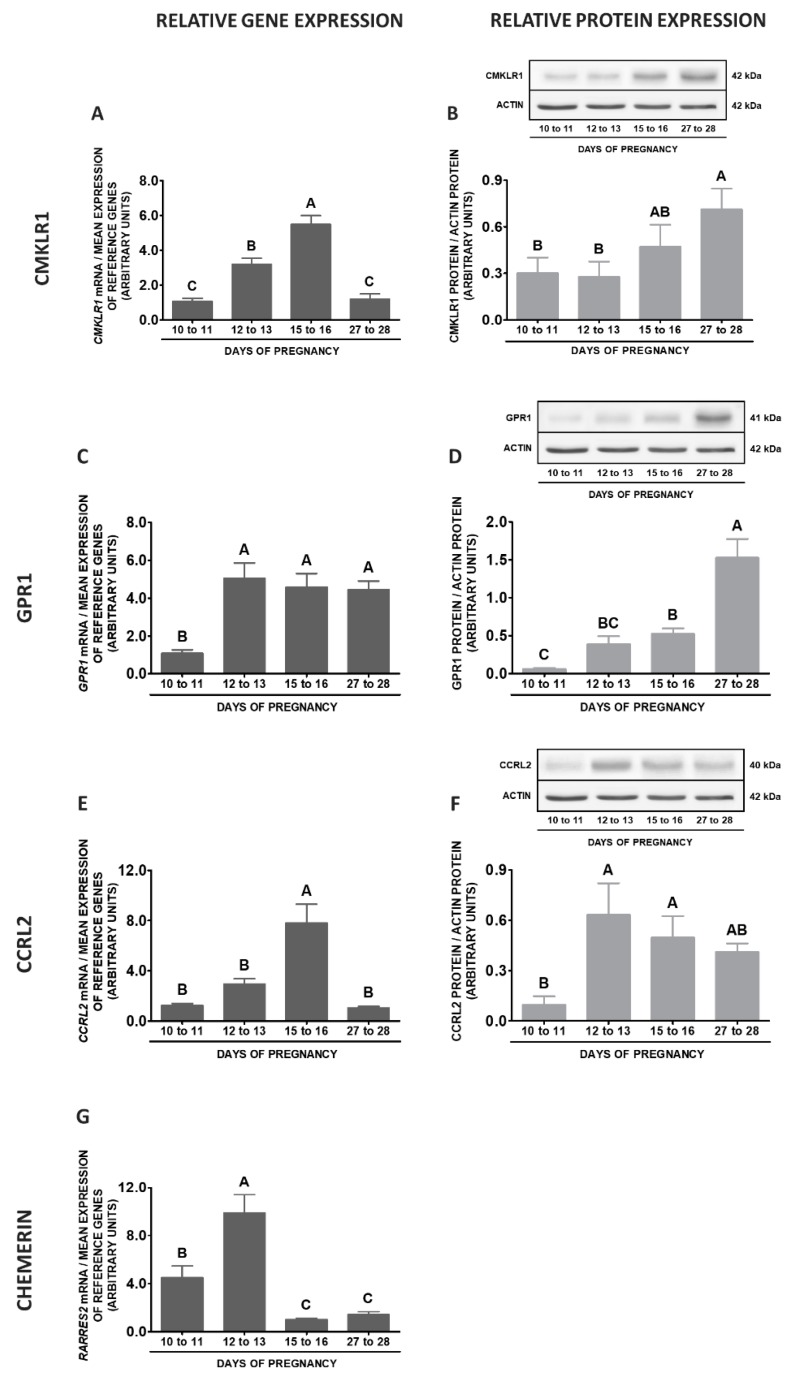
Chemerin system expression in the porcine mediobasal hypothalamus (MBH) during early pregnancy. Gene and protein expression of chemokine-like receptor 1 (CMKLR1; **A** and **B**), G protein-coupled receptor 1 (GPR1; **C** and **D**) and C-C motif chemokine receptor-like 2 (CCRL2; **E** and **F**), as well as chemerin (*RARRES2*) mRNA content (**G**) in the porcine MBH on days: 10 to 11, 12 to 13, 15 to 16 and 27 to 28 of pregnancy. Gene expression was determined by qPCR. Protein expression was determined by Western blotting; upper panels: representative immunoblots; lower panels: densitometric analysis of CMKLR1/GPR1/CCRL2 proteins relative to actin protein. Representative actin blots are identical for all chemerin receptors though proteins have been analysed on different gels. Results are presented as means ± SEM (*n* = 5). Bars with different superscripts differ (one-way ANOVA at *p* < 0.05 followed by Tuckey *post-hoc* test at *p* < 0.05).

During pregnancy, the highest *CMKLR* gene expression was observed on days 15 to 16, lower on days 12 to 13, and the lowest on days 10 to 11 and 27 to 28. CMKLR1 protein contents in MBH were greater on days 27 to 28 when compared to days 10 to 11 and 12 to 13 of gestation (*p* < 0.05; Figure 6A,B). The lowest *GPR1* gene expression was noted on days 10 to 11 in relation to other studied stages of pregnancy. Similarly, the lowest protein contents were observed on days 10 to 11, higher on days 15 to 16, and the highest on days 27 to 28 of pregnancy (*p* < 0.05; Figure 6C,D). The expression of *CCRL2* gene was higher on days 15 to 16 related to other studied periods of gestation, whereas protein contents were elevated on days 12 to 13 and 15 to 16 in comparison to days 10 to 11 (*p* < 0.05; Figure 6E,F). The highest expression of *RARRES2* gene was observed on days 12 to 13, lower on days 10 to 11, and the lowest on days 15 to 16 and 27 to 28 of pregnancy (*p* < 0.05; Figure 6G).

### 2.4. CHEM System Gene and Protein Expression in MBH—Pregnancy vs. Oestrous Cycle

**Figure 7:**
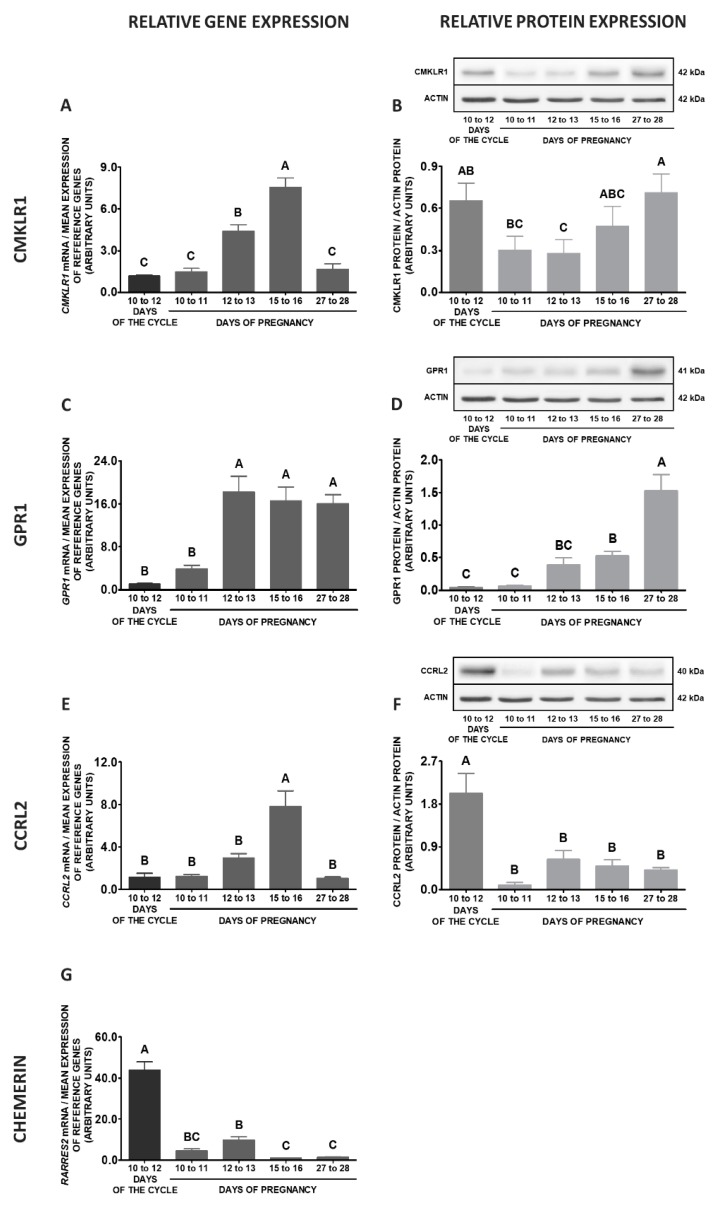
Chemerin system expression in the porcine mediobasal hypothalamus (MBH)—pregnancy vs. oestrous cycle. Gene and protein expression of chemokine-like receptor 1 (CMKLR1; **A** and **B**), G protein-coupled receptor 1 (GPR1; **C** and **D**) and C-C motif chemokine receptor-like 2 (CCRL2; **E** and **F**), as well as chemerin (*RARRES2*) mRNA content (**G**) in the porcine MBH on days: 10 to 11, 12 to 13, 15 to 16 and 27 to 28 of pregnancy, and on days 10 to 12 of the oestrous cycle. Gene expression was determined by qPCR. Protein expression was determined by Western blotting; upper panels: representative immunoblots; lower panels: densitometric analysis of CMKLR1/GPR1/CCRL2 proteins relative to actin protein. Representative actin blots are identical for all chemerin receptors though proteins have been analysed on different gels. Results are presented as means ± SEM (*n* = 5). Bars with different superscripts differ (one-way ANOVA at *p* < 0.05 followed by Tuckey *post-hoc* test at *p* < 0.05).

Comparing the studied stages of early pregnancy and days 10 to 12 of the oestrous cycle, significantly higher expression of *CMKLR1* gene in MBH was observed on days 15 to 16 and 12 to 13 of gestation in relation to days 10 to 12 of the oestrous cycle. The protein concentrations of this receptor were lower on days 12 to 13 of gestation, compared to days 10 to 12 of the cycle (*p* < 0.05; Figure 7A,B). The expression of the *GPR1* gene was enhanced on days 12 to 13, 15 to 16 and 27 to 28 of pregnancy when compared to days 10 to 12 of the cycle. Significantly higher GPR1 protein contents were observed on days 27 to 28 and 15 to 16 of gestation in relation to days 10 to 12 of the oestrous cycle (*p* < 0.05; Figure 7C,D). The expression of the *CCRL2* gene was elevated on days 15 to 16 of pregnancy compared to the cycle. The protein contents of this receptor were higher on days 10 to 12 of the cycle compared to the studied stages of pregnancy (*p* < 0.05; Figure 7E,F). Higher levels of *RARRES2* mRNA contents were observed on days 10 to 12 of the cycle than during the studied stages of gestation (*p* < 0.05; Figure 7G).

### 2.5. CHEM System Gene and Protein Expression in POA during the Oestrous Cycle

**Figure 8:**
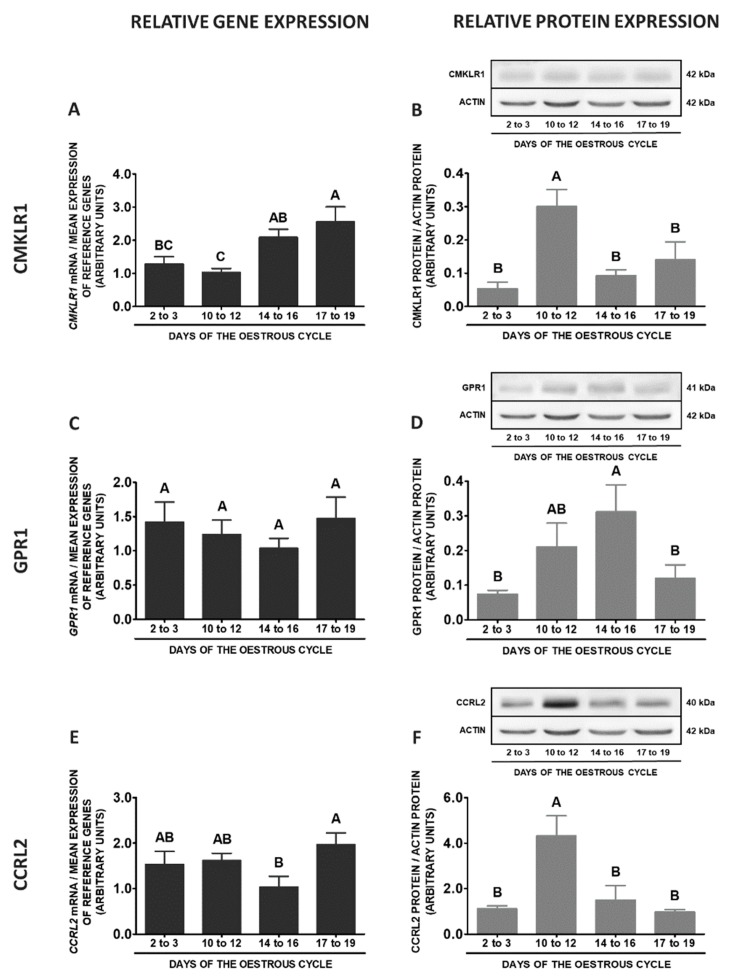

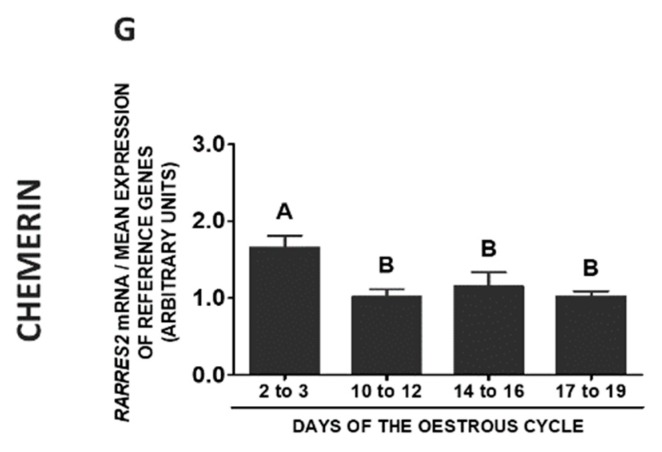
Chemerin system expression in the porcine preoptic area (POA) during the oestrous cycle. Gene and protein expression of chemokine-like receptor 1 (CMKLR1; **A** and **B**), G protein-coupled receptor 1 (GPR1; **C** and **D**) and C-C motif chemokine receptor-like 2 (CCRL2; **E** and **F**), as well as chemerin (*RARRES2*) mRNA content (**G**) in the porcine POA on days: 2 to 3, 10 to 12, 14 to 16 and 17 to 19 of the oestrous cycle. Gene expression was determined by qPCR. Protein expression was determined by Western blotting; upper panels: representative immunoblots; lower panels: densitometric analysis of CMKLR1/GPR1/CCRL2 proteins relative to actin protein. Representative actin blots are identical for all chemerin receptors though proteins have been analysed on different gels. Results are presented as means ± SEM (*n* = 5). Bars with different superscripts differ (one-way ANOVA at *p* < 0.05 followed by Tuckey *post-hoc* test at *p* < 0.05).

In the porcine POA, the highest *CMKLR1* gene expression was observed on days 17 to 19 of the cycle when compared to days 2 to 3 and 10 to 12, whereas the lowest on days 10 to 12 of the cycle in relation to days 14 to 16 and 17 to 19. The highest protein contents of this receptor were noted on days 10 to 12 compared to other phases of the cycle (*p* < 0.05; Figure 8A,B). Although the differences in *GPR1* gene expression between the studied phases of the oestrous cycle were negligible, the higher protein concentrations were observed on days 14 to 16 compared to days 2 to 3 and 17 to 19 of the cycle (*p* < 0.05; Figure 8C,D). The expression of the *CCRL2* gene was higher on days 17 to 19 than on days 14 to 16 of the oestrous cycle. The protein concentrations of CCRL2 were higher on days 10 to 12 compared to other phases of the cycle (*p* < 0.05; Figure 8E,F). In POA, higher *RARRES2* gene expression was noted on days 2 to 3 of the cycle than in the other studied phases of pregnancy (*p* < 0.05; Figure 8G).

### 2.6. CHEM System Gene and Protein Expression in POA during Pregnancy

**Figure 9:**
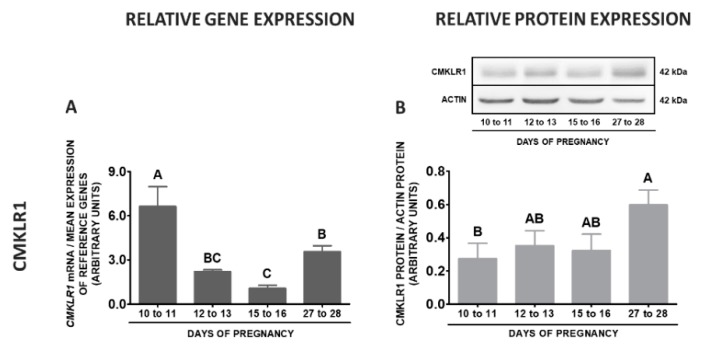

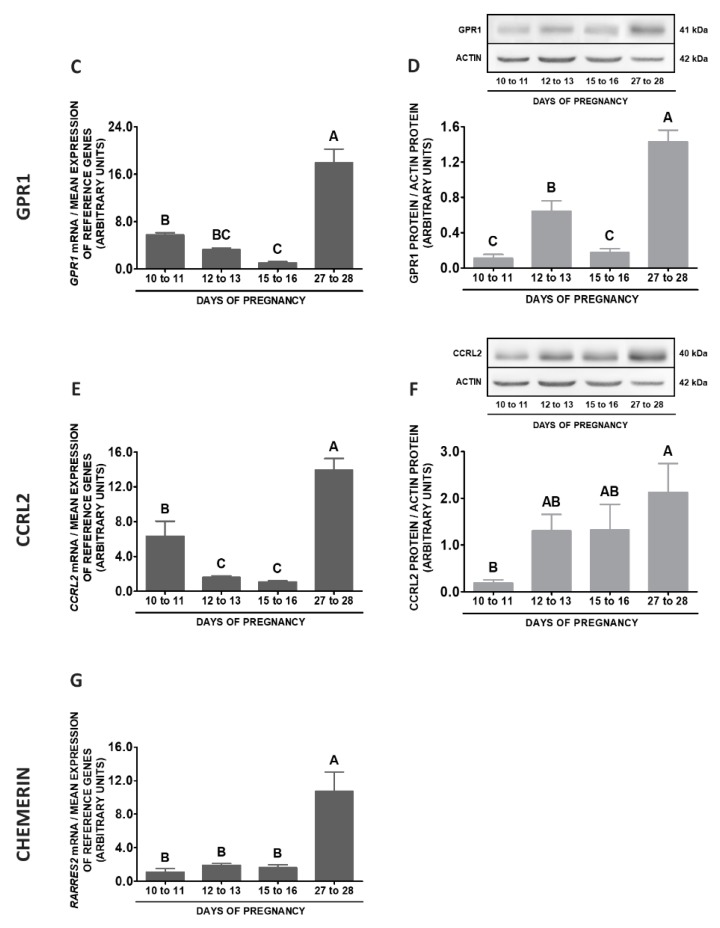
Chemerin system expression in the porcine preoptic area (POA) during early pregnancy. Gene and protein expression of chemokine-like receptor 1 (CMKLR1; **A** and **B**), G protein-coupled receptor 1 (GPR1; **C** and **D**) and C-C motif chemokine receptor-like 2 (CCRL2; **E** and **F**), as well as chemerin (*RARRES2*) mRNA content (**G**) in the porcine POA on days: 10 to 11, 12 to 13, 15 to 16 and 27 to 28 of pregnancy. Gene expression was determined by qPCR. Protein expression was determined by Western blotting; upper panels: representative immunoblots; lower panels: densitometric analysis of CMKLR1/GPR1/CCRL2 proteins relative to actin protein. Representative actin blots are identical for all chemerin receptors though proteins have been analysed on different gels. Results are presented as means ± SEM (*n* = 5). Bars with different superscripts differ (one-way ANOVA at *p* < 0.05 followed by Tuckey *post-hoc* test at *p* < 0.05).

During early pregnancy in POA, the highest *CMKLR1* gene expression was observed on days 10 to 11, lower on days 27 to 28, and the lowest on days 15 to 16 when compared to days 10 to 11 and 27 to 28 of gestation. Protein contents of this receptor were higher on days 27 to 28 related to days 10 to 11 of pregnancy (*p* < 0.05; Figure 9A,B). The contents of the *GPR1* mRNA were the highest on days 27 to 28 compared to other stages of pregnancy, whereas the lowest on days 15 to 16 in relation to days 10 to 11 and 27 to 28 of gestation. Similarly, the highest protein concentrations of GPR1 were noted on days 27 to 28, lower on days 12 to 13 and the lowest on days 10 to 11 and 15 to 16 of pregnancy (*p* < 0.05; Figure 9C,D). The expression of the *CCRL2* gene in this tissue was the highest on days 27 to 28, lower on days 10 to 11, whereas the lowest on days 12 to 13 and 15 to 16 of gestation. The protein concentrations of this receptor were higher on days 27 to 28 than on days 10 to 11 of pregnancy (*p* < 0.05; Figure 9E,F). The expression of the *RARRES2* gene was much more pronounced on days 27 to 28 than in other studied stages of pregnancy (*p* < 0.05; Figure 9G).

### 2.7. CHEM System Gene and Protein Expression in POA—Pregnancy vs. Oestrous Cycle

**Figure 10:**
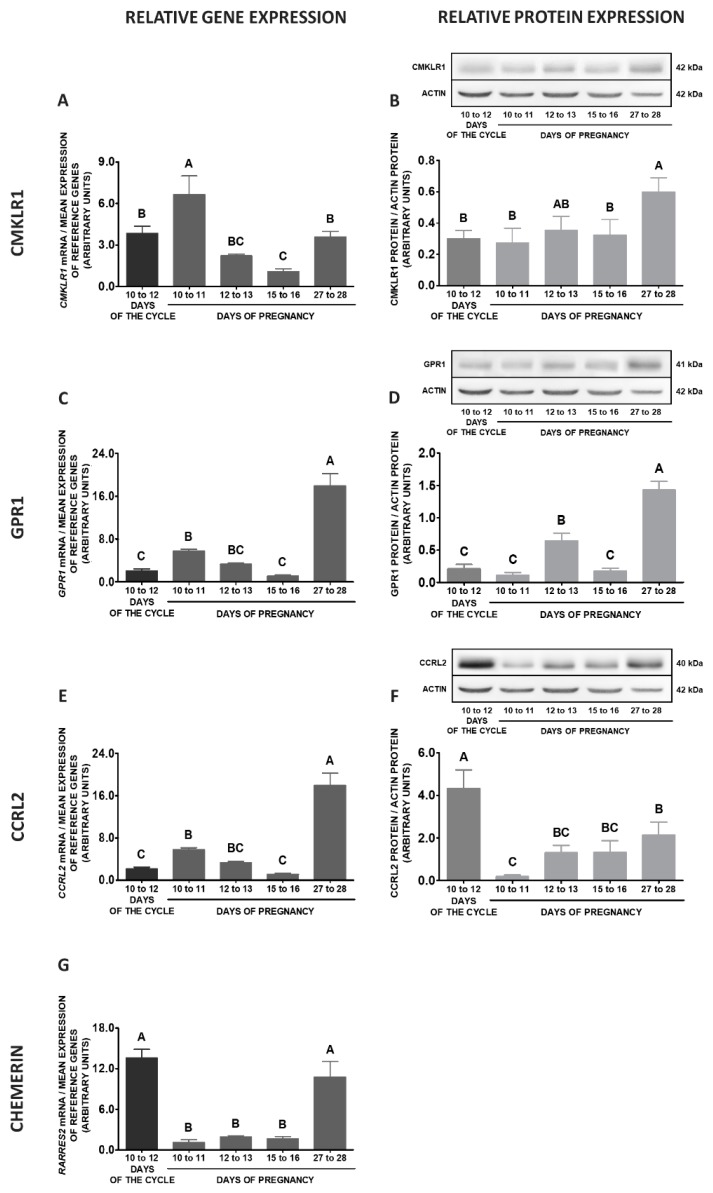
Chemerin system expression in the porcine preoptic area (POA)—pregnancy vs. oestrous cycle. Gene and protein expression of chemokine-like receptor 1 (CMKLR1; **A** and **B**), G protein-coupled receptor 1 (GPR1; **C** and **D**) and C-C motif chemokine receptor-like 2 (CCRL2; **E** and **F**), as well as chemerin (*RARRES2*) mRNA content (**G**) in the porcine POA on days: 10 to 11, 12 to 13, 15 to 16 and 27 to 28 of pregnancy, and on days 10 to 12 of the oestrous cycle. Gene expression was determined by qPCR. Protein expression was determined by Western blotting; upper panels: representative immunoblots; lower panels: densitometric analysis of CMKLR1/GPR1/CCRL2 proteins relative to actin protein. Representative actin blots are identical for all chemerin receptors though proteins have been analysed on different gels. Results are presented as means ± SEM (*n* = 5). Bars with different superscripts differ (one-way ANOVA at *p* < 0.05 followed by Tuckey *post-hoc* test at *p* < 0.05).

Comparing the studied stages of pregnancy and days 10 to 12 of the oestrous cycle, higher *CMKLR1* gene expression was noted on days 10 to 11 of pregnancy, whereas lower on days 15 to 16 in relation to days 10 to 12 of the cycle. Protein concentrations of this receptor were higher on days 27 to 28 of pregnancy compared to days 10 to 12 of the cycle (*p* < 0.05; Figure 10A,B). The expression of *GPR1* gene during the mid-luteal phase was lower than on days 27 to 28 and 10 to 11 of pregnancy. Protein contents of this receptor on days 10 to 12 of the cycle were lower than on days 27 to 28 of pregnancy and days 12 to 13 of pregnancy (*p* < 0.05; Figure 10C,D). Higher contents of *CCRL2* mRNA were observed on days 27 to 28 of and 10 to 11 of gestation in relation to days 10 to 12 of the cycle. The highest protein concentrations of this receptor were noted on days 10 to 12 of the cycle in comparison to the studied stages of gestation (*p* < 0.05; Figure 10E,F). An elevated expression of the *RARRES2* gene was observed on days 10 to 12 of the cycle in relation to days 10 to 11, 12 to 13 and 15 to 16 of gestation (*p* < 0.05; Figure 10G).

### 2.8. CHEM System Gene and Protein Expression in SME during the Oestrous Cycle

**Figure 11:**
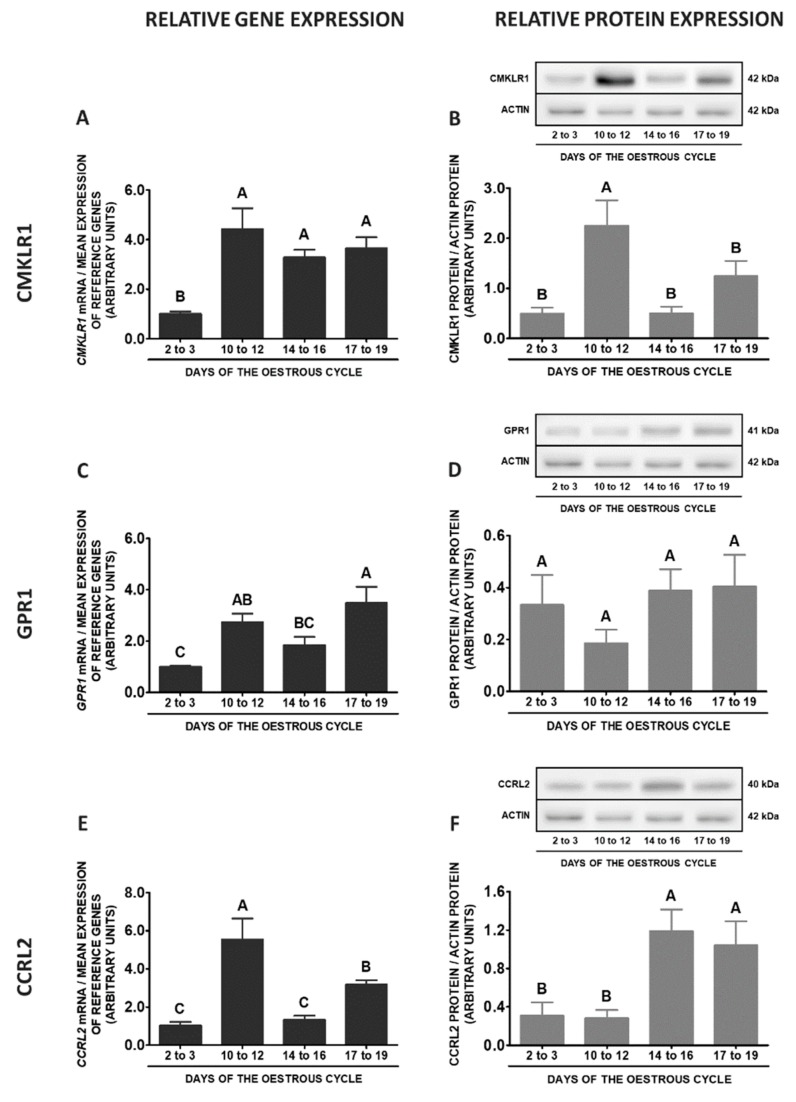

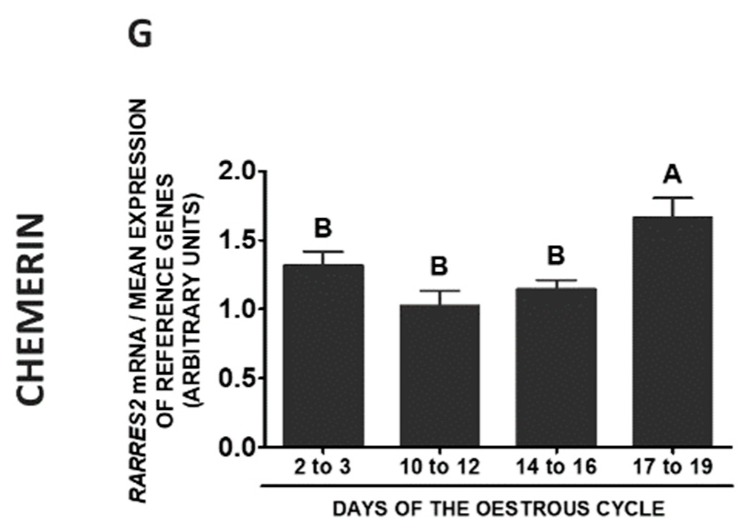
Chemerin system expression in the porcine stalk median eminence (SME) during the oestrous cycle. Gene and protein expression of chemokine-like receptor 1 (CMKLR1; **A** and **B**), G protein-coupled receptor 1 (GPR1; **C** and **D**) and C-C motif chemokine receptor-like 2 (CCRL2; **E** and **F**), as well as chemerin (*RARRES2*) mRNA content (**G**) in the porcine SME on days: 2 to 3, 10 to 12, 14 to 16 and 17 to 19 of the oestrous cycle. Gene expression was determined by qPCR. Protein expression was determined by Western blotting; upper panels: representative immunoblots; lower panels: densitometric analysis of CMKLR1/GPR1/CCRL2 proteins relative to actin protein. Representative actin blots are identical for all chemerin receptors though proteins have been analysed on different gels. Results are presented as means ± SEM (*n* = 5). Bars with different superscripts differ (one-way ANOVA at *p* < 0.05 followed by Tuckey *post-hoc* test at *p* < 0.05).

During the oestrous cycle, the expression of the *CMKLR1* gene in SME was significantly decreased on days 2 to 3 in relation to other phases of the cycle. However, the protein concentrations of this receptor were increased only on days 10 to 12 compared to other phases of the cycle (*p* < 0.05; Figure 11A,B). The highest *GPR1* mRNA contents were noted on days 17 to 19 compared to days 2 to 3 and 14 to 16, whereas the lowest on days 2 to 3 in relation to days 10 to 12 and 17 to 19 of the cycle. The differences in this receptor protein concentrations during the oestrous cycle were negligible (*p* < 0.05; Figure 11C,D). The expression of the *CCRL2* gene was the highest on days 10 to 12, lower on days 17 to 19, whereas the lowest on days 2 to 3 and 14 to 16 of the cycle. An elevated contents of CCRL2 protein were observed on days 14 to 16 and 17 to 19 in relation to days 2 to 3 and 10 to 12 of the cycle (*p* < 0.05; Figure 11E,F). The expression of *RARRES2* gene was enhanced on days 17 to 19 when compared to other studied stages of the cycle (*p* < 0.05; Figure 11G).

### 2.9. CHEM System Gene and Protein Expression in SME during Pregnancy

**Figure 12:**
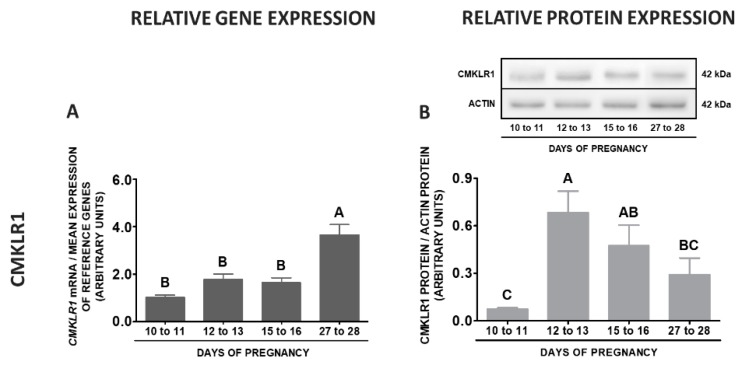

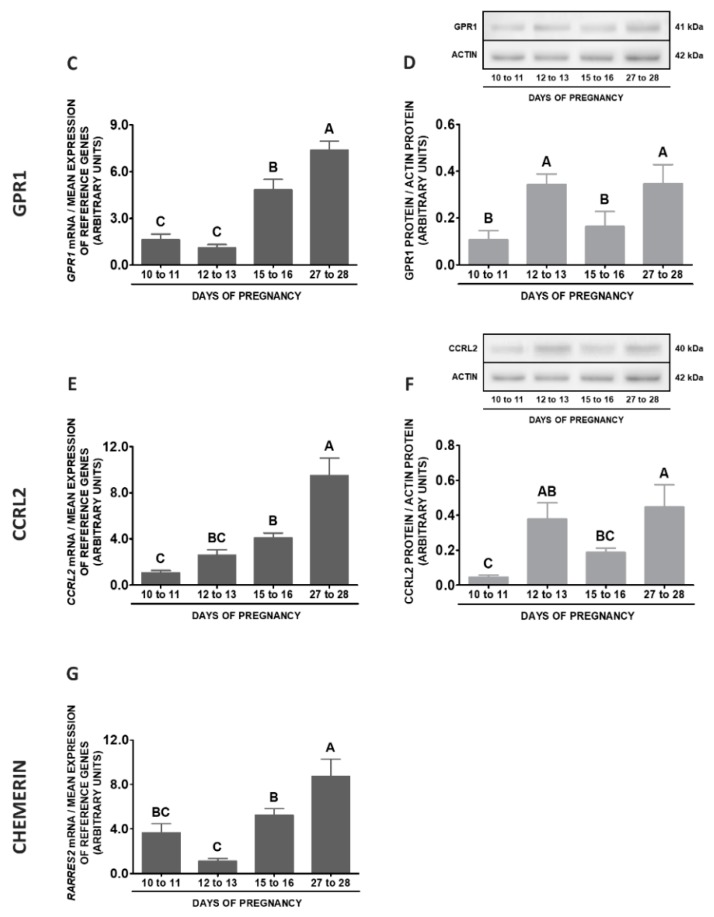
Chemerin system expression in the porcine stalk median eminence (SME) during early pregnancy. Gene and protein expression of chemokine-like receptor 1 (CMKLR1; **A** and **B**), G protein-coupled receptor 1 (GPR1; **C** and **D**) and C-C motif chemokine receptor-like 2 (CCRL2; **E** and **F**), as well as chemerin (*RARRES2*) mRNA content (**G**) in the porcine SME on days: 10 to 11, 12 to 13, 15 to 16 and 27 to 28 of pregnancy. Gene expression was determined by qPCR. Protein expression was determined by Western blotting; upper panels: representative immunoblots; lower panels: densitometric analysis of CMKLR1/GPR1/CCRL2 proteins relative to actin protein. Representative actin blots are identical for all chemerin receptors though proteins have been analysed on different gels. Results are presented as means ± SEM (*n* = 5). Bars with different superscripts differ (one-way ANOVA at *p* < 0.05 followed by Tuckey *post-hoc* test at *p* < 0.05).

During pregnancy, an elevated levels of *CMKLR1* mRNA were observed on days 27 to 28 comparing to other stages of early pregnancy. Protein concentrations of this receptor were the highest on days 12 to 13 in relation to days 10 to 11 and 27 to 28, and the lowest on days 10 to 11 compared to days 12 to 13 and 15 to 16 of pregnancy (*p* < 0.05; Figure 12A,B). The expression of the *GPR1* gene was the highest on days 27 to 28 of pregnancy, lower on days 15 to 16, whereas the lowest on days 10 to 11 and 12 to 13 of gestation. The concentrations of GPR1 protein were elevated on days 12 to 13 and 27 to 28 of pregnancy in relation to the remaining stages of pregnancy (*p* < 0.05; Figure 12C,D). The highest *CCRL2* gene expression was noted on days 27 to 28, whereas the lowest on days 10 to 11 when compared to days 15 to 16 and 27 to 28 of pregnancy. The protein contents of this receptor were the highest on days 27 to 28 in relation to days 10 to 11 and 15 to 16, whereas the lowest on days 10 to 11 compared to days 12 to 13 and 27 to 28 of pregnancy (*p* < 0.05; Figure 12E,F). The highest expression of the *RARRES2* gene was observed on days 27 to 28 of gestation, whereas the lowest on days 12 to 13 in relation to days 15 to 16 and 27 to 28 of pregnancy (*p* < 0.05; Figure 12G).

### 2.10. CHEM System Gene and Protein Expression in SME—Pregnancy vs. Oestrous Cycle

**Figure 13:**
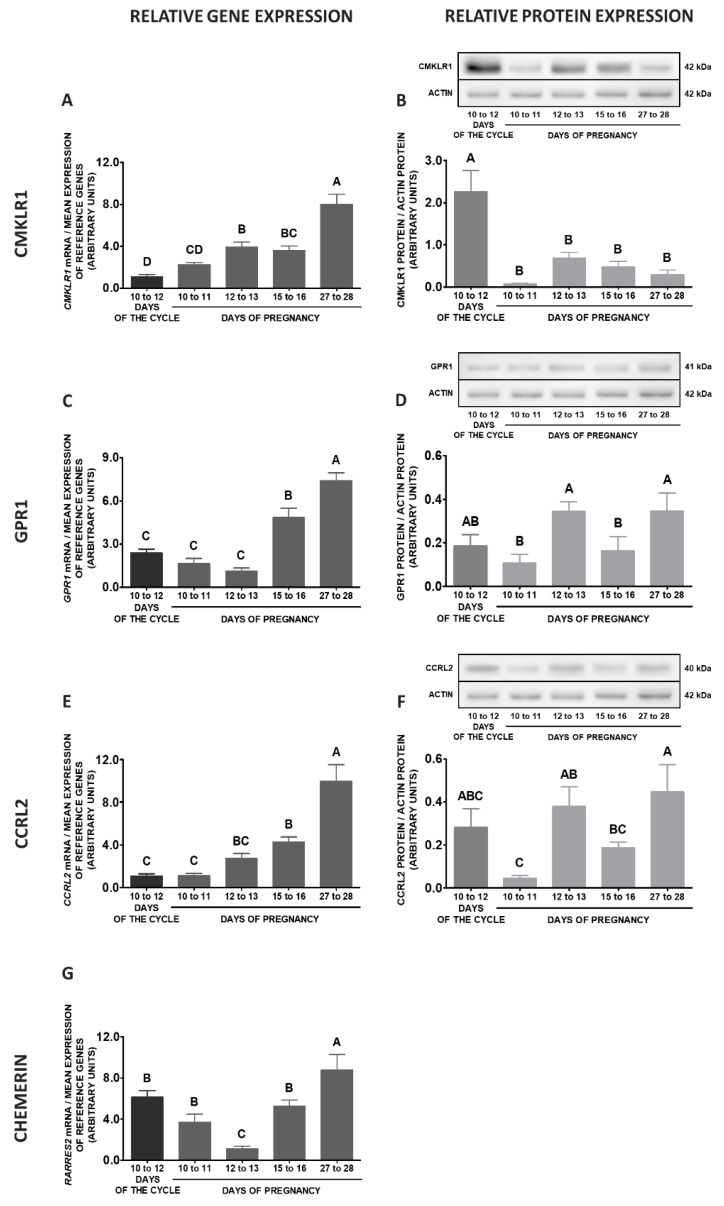
Chemerin system expression in the porcine stalk median eminence (SME)—pregnancy vs. oestrous cycle. Gene and protein expression of chemokine-like receptor 1 (CMKLR1; **A** and **B**), G protein-coupled receptor 1 (GPR1; **C** and **D**) and C-C motif chemokine receptor-like 2 (CCRL2; **E** and **F**), as well as chemerin (*RARRES2*) mRNA content (**G**) in the porcine SME on days: 10 to 11, 12 to 13, 15 to 16 and 27 to 28 of pregnancy, and on days 10 to 12 of the oestrous cycle. Gene expression was determined by qPCR. Protein expression was determined by Western blotting; upper panels: representative immunoblots; lower panels: densitometric analysis of CMKLR1/GPR1/CCRL2 proteins relative to actin protein. Representative actin blots are identical for all chemerin receptors though proteins have been analysed on different gels. Results are presented as means ± SEM (*n* = 5). Bars with different superscripts differ (one-way ANOVA at *p* < 0.05 followed by Tuckey *post-hoc* test at *p* < 0.05).

Comparing the studied stages of pregnancy and days 10 to 12 of the oestrous cycle, significantly lower *CMKLR1* gene expression was noted on days 10 to 12 of the oestrous cycle in relation to days 12 to 13, 15 to 16 and 27 to 28 of gestation. Protein concentrations of this receptor were significantly higher on days 10 to 12 of the cycle compared to the studied stages of pregnancy (*p* < 0.05; Figure 13A,B). The expression of the *GPR1* gene during the mid-luteal phase of the cycle was lower than on days 15 to 16 and 27 to 28 of gestation. Protein contents of this receptor on days 10 to 12 of the oestrous cycle did not differ from the studied stages of gestation (*p* < 0.05; Figure 13C,D). The gene expression of *CCRL2* was lower on days 10 to 12 of the cycle compared to days 15 to 16 and 27 to 28 of pregnancy. Protein contents of this receptor on days 10 to 12 of the oestrous cycle did not differ from the studied stages of gestation (*p* < 0.05; Figure 13E,F). The gene expression of *RARRES2* on days 10 to 12 of the cycle was higher than on days 12 to 13 of pregnancy, whereas lower than on days 27 to 28 of gestation (*p* < 0.05; Figure 13G).

### 2.11. CHEM Concentrations in the Blood Plasma

**Figure 14:**
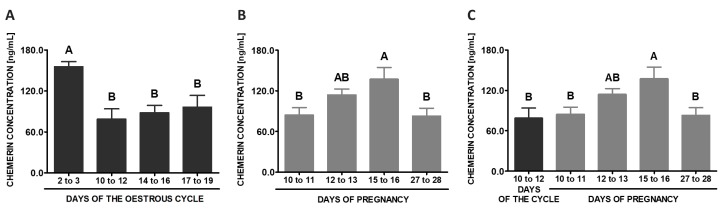
Chemerin concentrations in the porcine blood plasma. Concentrations of chemerin in the porcine blood plasma was determined during the oestrous cycle on days: 2 to 3, 10 to 12, 14 to 16 and 17 to 19 (**A**), during early pregnancy on days: 10 to 11, 12 to 13, 15 to 16 and 27 to 28 (**B**) and compared between early pregnancy and days 10 to 12 of the oestrous cycle (**C**). The hormone content in blood plasma was evaluated using ELISA. Results are presented as means ± SEM (*n* = 5). Bars with different superscripts differ (one-way ANOVA at *p* < 0.05 followed by Tuckey *post-hoc* test at *p* < 0.05).

During the oestrous cycle, the highest concentrations of CHEM in the blood plasma were observed on days 2 to 3 (*p* < 0.05; Figure 14A). During early pregnancy, significantly higher CHEM contents in the plasma were noted on days 15 to 16 comparing to days 10 to 11 and 27 to 28 (*p* < 0.05; Figure 14B). Comparing the studied stages of early pregnancy and days 10 to 12 of the cycle, significantly higher concentrations of CHEM in the blood plasma were observed on days 15 to 16 of gestation in relation to the cycle (*p* < 0.05; Figure 14C).

## 3. Discussion

The present study was the first experiment to report the expression of CHEM and its receptors genes and proteins in the porcine hypothalamic structures, MBH, POA and SME, responsible for GnRH production and secretion, during the oestrous cycle and early pregnancy. In our immunohistochemical analyses, CHEM and its receptors were localised in the porcine paraventricular nucleus, which is the part of mediobasal hypothalamus and in the preoptic region during the oestrous cycle and early pregnancy. In the diagonal band of Broca, a part of preoptic region, very high immunoreactivity of GPR1 was observed. To date, the *RARRES2* gene was expressed in the hypothalamus of mice and rats [10,11]. In the brain of mice, *RARRES2* mRNA expression was restricted to the dorsal ventricular wall of the anterior, medial and posterior hypothalamus [10]. In turn, in the brain of rats, *RARRES2* mRNA was present in the tanycytes and ependymal cells layer lining the ventral third ventricle and SME of the hypothalamus, and *CMKLR1* transcript was found in the prefrontal cortex, hippocampus, cerebellum, ependymal cell layer and SME [11,12]. The transcripts of *RARRES2* were also present in the hypothalamus of baboons and chimpanzees [23]. GPR1 is expressed abundantly in the human brain, such as hippocampus, glioblastoma cells, brain-derived fibroblast-like cells lines and microglia [13]. In the ependymal cell layer and SME of rats, the gene expression of third CHEM receptor, *CCRL2* is also localised [11]. After CHEM injection, the expression of the cytoskeletal protein vimentin, a marker for estimating hypothalamic plasticity, was increased in the ependymal cell region of rats. It has been suggested that CHEM plays an important role in the structural remodelling of the hypothalamus [11]. The above and our studies indicate that the hypothalamus is capable of synthesising the discussed adipokine. The local production of CHEM and the presence of specific receptors suggest that the hormone exerts a direct effect on the hypothalamus with possible autocrine and/or paracrine action of CHEM in the brain.

CHEM is also delivered to the central nervous system (CNS) with the blood. It was found in the cerebrospinal fluid (CSF) of humans and the CHEM-158K protein is the dominant CHEM isoform. The total levels of cleaved and noncleaved CHEM were significantly lower in the CSF samples from patients with a variety of CNS diseases than in the plasma. In turn, in normal human plasma, the prochemerin form (CHEM-163S) dominated, whereas CHEM-158K represented only a small percentage of the total CHEM, and CHEM-157S was barely detectable. Thus, under normal conditions, the majority of circulating CHEM exists in the relatively inactive prochemerin form and requires proteolytic processing to bioactive CHEM isoforms. Only about 18% of CHEM is cleaved in normal plasma, whereas in CSF samples the fraction ranges about 50%. It has been implied that considerable proteolysis of CHEM is taking place in this extravascular compartment. Moreover, the differences between the total amount of CHEM as well as the extent of its cleavage that was found in the CSF supported the hypothesis of local CHEM synthesis and processing [24].

In the human blood, relatively high levels of CHEM (100–200 ng/mL) have been detected [25]. Plasma CHEM levels are correlated with BMI, body mass and adipose tissue mass. A statistically significant correlation has also been found between the adipokine level, and age and sex. Higher CHEM levels were found in women and older subjects than in men and younger subjects [3]. Our present studies indicate the higher levels of porcine plasma CHEM during the early-luteal phase (oestradiol (E_2_) and progesterone (P_4_) plasma concentrations are at moderate levels) than during the mid- and late-luteal phases (P_4_ levels are high in the porcine blood) and follicular phase (E_2_ levels are high in the porcine blood) of the oestrous cycle. The above results suggest that sex hormones, particularly gonadal steroids, may affect the synthesis of CHEM. Such a correlation has been documented for androgens increasing the levels of CHEM in women [19,26] and female rats [17]. A stimulatory effect of insulin on the synthesis of CHEM and its plasma levels was also demonstrated [19]. The levels of CHEM also change throughout pregnancy and between pregnancy and the oestrous cycle. In the present study, during the beginning of implantation, porcine plasma CHEM contents were enhanced compared to the period of embryos migration within the uterus, end of implantation and mid-luteal phase of the oestrous cycle. In the blood of pregnant women, CHEM levels were significantly higher than in the non-pregnant ones [27]. Moreover, plasma levels of CHEM were significantly elevated during late gestation when compared to the early gestation of humans [27], but the reverse correlation was found in rats [28]. This may indicate species–specific differences in the activity of CHEM.

To our knowledge, this is the first study that compares CHEM system genes expression and proteins concentrations in the hypothalamus within the oestrous cycle and early pregnancy and between these physiological periods. Our obtained results indicate that CHEM expression levels were determined by the stage of the oestrous cycle and early pregnancy and these results confirmed the hypothesis that steroid hormones may regulate the synthesis of CHEM. The expression of CMKLR1 and CCLR2 proteins in most hypothalamic structures increased during the mid-luteal phase of the oestrous cycle, which seems to suggest the up-regulating effect of P_4_. In POA, the expression of GPR1 protein was also stimulated during the luteal phase of the cycle. However, GPR1 protein contents in MBH were enhanced during the follicular phase, when plasma levels of oestrogens are very high. In addition, the concentrations of CHEM receptors proteins in the porcine hypothalamic structures generally increase with the advancement of pregnancy from days 10 to days 28. In our previous study, in the porcine peripheral blood plasma from days 10 to days 28 of pregnancy, a statistically significant decrease in the mean of P_4_ levels was observed [29]. These results may suggest an inverse pattern between the expression of CHEM receptors and the levels of P_4_ during pregnancy. The role of steroids in the regulation of CHEM system was also investigated in humans and the results are also unclear. The findings of an inverse association of E_2_ and CHEM levels have been observed in a clinical study comprising both lean and overweight men and women [30], but no significant effects of steroids on CHEM protein production in the control human subcutaneous adipose tissue explants were reported [19]. It cannot be ruled out that other hormones regulating the oestrous cycle, such as the pituitary luteinizing hormone (LH), may be involved in the regulation of CHEM expression. Men with elevated LH levels had lower CHEM contents in relation to those with the normal range of LH [31]. Further studies would be recommended to identify the exact mechanisms of the relationship between the contents of CHEM and steroid/other hormones. Moreover, in our study, we have also observed the higher GPR1 and lower CCRL2 protein concentrations in the porcine MBH and POA during the early pregnancy versus the oestrous cycle. These findings supported the idea that CHEM/GPR1/CCRL2 could be the one of the regulators of pregnancy at the hypothalamic level. However, the possible roles of CHEM in the maintenance of pregnancy should be investigated in further studies.

Some lines of evidence suggest that CHEM regulates hypothalamic hormones secretion connected with feeding behaviour. A direct stimulating effect of CHEM on hypothalamic agouti-related peptide and pro-opiomelanocortin gene expression was observed in rats after injecting a single dose of CHEM (8 µg/kg) in the arcuate nucleus [32]. These results are in good agreement with reports indicating that the administration of the major dosage of CHEM (16 g/kg) increased mRNA contents of both cocaine and amphetamine-regulated transcript (CART) and neuropeptide Y [15]. The above results confirmed the role of CHEM in the regulation of hypothalamic modulators. However, there is a lack of data on the role of CHEM on the reproductive functions at the brain level, in the hypothalamus and pituitary through the regulation of GnRH, LH and follicle-stimulating hormone production. Several studies have reported the direct role of CHEM on the gonads. The expression of the *RARRES2* gene was found in the ovaries of women [8], rodents [2,9] and cows [33]. Mammalian ovaries are also sensitive to CHEM: CMKLR1 expression was identified in the ovaries of women [8] and rats [9], while CMKLR1, GPR1 and CCLR2 expression was noticed in the ovaries of cows [33]. A clear effect of CHEM on ovarian steroidogenesis was demonstrated and reflected by the inhibition of FSH-induced secretion of P_4_ and E_2_ by granulosa cells in rats [9,18], cows [33] and the insulin-like growth factor 1-induced secretion of both steroids in granulosa cells in women [8]. The presence of the CHEM system in hypothalamic structures responsible for GnRH synthesis and secretion observed in our study implies that the discussed hormone regulates GnRH generation. Therefore, it cannot be excluded that the adipokine can affect the gonads indirectly via effects on the central hypothalamic–pituitary–gonadal axis, i.e., by affecting the GnRH hypothalamic neurons and/or the gonadotrophs of the pituitary; however, this hypothesis needs to be verified.

## 4. Materials and Methods

### 4.1. Experimental Animals and Tissue Collection

Experimental animals were mature gilts (Large White × Polish Landrace), about 7–8 months old, weighing 120–130 kg, descended from private breeding. All individuals were given access to water and forage ad libitum. Forty animals were assigned to one of eight experimental groups (*n* = 5 in each group) as follows: days 2 to 3 (early luteal phase, the presence of corpora hemorrhagica), 10 to 12 (mid-luteal phase, the phase when the corpus luteum activity is high and similar to that noted during pregnancy), 14 to 16 (late luteal phase, the period of luteolysis) and 17 to 19 (follicular phase) of the oestrous cycle, and 10 to 11 (migration of the embryos within the uterus), 12 to 13 (maternal recognition of pregnancy), 15 to 16 (beginning of implantation) and 27 to 28 (end of implantation) of pregnancy. Cyclic gilts were monitored daily for an oestrous behaviour in the presence of a boar. The day of the onset of the second oestrous was designated as day 0 of the oestrous cycle. The phase of the oestrous cycle was also confirmed based on ovaries morphology [34]. In the case of pregnant pigs, the insemination was performed on days 1 to 2 of the oestrous cycle. Pregnancy was confirmed by the presence and morphology of conceptuses. On days 10 to 11 and 12 to 13 of pregnancy, conceptuses were obtained by the flushing of uterine horns with 20 mL of sterile phosphate-buffered saline (PBS), whereas on days 15 to 16 and 27 to 28 of pregnancy, by dissection from the endometrium. Within a few minutes after slaughter, blood samples were collected and the hypothalamus was dissected. Blood samples were placed into heparinised tubes, centrifuged (2500× *g*, 15 min, 4 °C) and the obtained plasma was stored at −80 °C. Each hypothalamic block was divided into: MBH, POA and SME as described by Sesti and Britt [35]. The mediobasal hypothalamus was defined as a block of tissue bound rostrally by the optic chiasma, caudally by mammillary body, laterally by the hypothalamic sulci and dorsally by a cut 5 mm deep. POA was limited rostrally approximately 5 mm anterior to the optic chiasma and caudally by the rostral border of MBH. The stalk median eminence was easily detached from the MBH and was cut away at its junction with the pituitary gland. Preoptic areas and MBH from days 15 to 16 of pregnancy and 10 to 12 of the cycle were divided in two parts, from which one half intended for immunofluorescent staining was placed in 4% buffered paraformaldehyde (pH = 7.4, 4 °C), whereas other halves, along with all the rest of the POAs, MBHs and SMEs were frozen in liquid nitrogen and stored at −80 °C until RNA and protein isolation.

Tissue samples were taken from animals intended for commercial slaughter and meat processing. All studies were conducted in accordance with ethical standards of the Animal Ethics Committee at the University of Warmia and Mazury in Olsztyn. The animals used in the study were reared and transported under conditions specified in the “Act on the protection of animals used for scientific or educational purposes” (Poland, 2015).

### 4.2. Fluorescent Immunohistochemistry of the Porcine Hypothalamus

The tissue blocks were fixed by immersion for 24 h in 4% buffered paraformaldehyde (pH = 7.4; 4 °C). Following fixation, the brains were washed in 0.1 M PBS and then cryoprotected for 3–5 days in graded solutions (19% and 30%) of sucrose (Sigma Aldrich, St. Louis, MO, USA) at 4 °C until they sank. The tissue blocks comprised of the hypothalamus and adjoining structures were frozen and cut into 18 μm thick cryostat coronal sections and stored at −80 °C. Frozen sections were processed for double-labelling immunofluorescence by means of primary antisera raised in different species. The sections were air-dried for 30 min, washed 3 times in cold PBS and incubated for 1 h with blocking buffer (0.1 M PBS, 10% normal donkey serum, 0.01% bovine serum albumin, 1% Tween, 0.05% thimerosal, 0.01% NaN3). Then sections were incubated overnight at room temperature with a solution of rabbit polyclonal antibodies against CMKLR1 (0.5 µg/uL, ab230442, Abcam, Cambridge, UK), or GPR1 (1.25 µg/uL, ab188977, Abcam, Cambridge, UK), or CCRL2 (0.67 µg/uL, ab85224, Abcam, Cambridge, UK), or CHEM (0.5 µg/uL, ab203040, Abcam, Cambridge, UK). In order to show the binding sites of the antisera with antigens, the sections were incubated with the Alexa Fluor 555 donkey anti-rabbit antibodies (0.5 µg/uL, A-31572, Molecular Probes, Eugene, OR, USA). After staining, scraps were washed in PBS and mounted with carbonate-buffered glycerol (pH 8.6) and cover slipped. All steps of the staining procedures were conducted in humid chambers at room temperature. Standard controls, i.e., the omission and replacement of primary antisera by non-immune sera were applied to test antibody and method specificity. The lack of any immunoreactions indicated specificity. The sections were analysed with an Olympus BX51 microscope equipped with a CC-12 digital camera (Soft Imaging System GMBH, Münster, Germany). Images were acquired with Cell-F software (Olympus GmbH, Hamburg, Germany).

### 4.3. Quantitative Real-Time PCR

Total RNA from MBH, POA and SME samples was isolated using the peqGold TriFast isolation system (Peqlabs, Erlangen, Germany). RNA quantity and quality were determined spectrometrically (Infinite M200 Pro, Tecan, Männedorf, Switzerland). First strand cDNA was synthesised using the Omniscript RT Kit (Qiagen, Hilden, Germany) in a total volume of 20 µL with 1 µg of RNA, 0.5 µg oligo(dT)15 (Roche, Basel, Switzerland) at 37 °C for 1 h and was terminated by the incubation at 93 °C for 5 min. Specific primer pairs used to amplify parts of *RARRES2*, *CMKLR1*, *GPR1*, *CCRL2*, ubiquitin C (*UBC*) and 18S ribosomal RNA (*18sRNA*) genes are detailed in Table 1. Specific primers for *RARRES2*, *CMKLR1*, *GPR1* and *CCRL2* were designed with the Primer Express 3.0 software (Life Technologies, Camarillo, CA, USA) and their specificities were confirmed by comparison of their sequences with the sequences of corresponding genes deposited in a GenBank database. Due to the calculation of the statistical significance of the match, the Basic Local Alignment Search Tool (BLAST) was used. Quantitative real-time PCR (qPCR) analysis was carried out using PCR System 7300 (Applied Biosystems Inc., Foster, CA, USA), as described previously by Smolinska et. al. [36]. Briefly, the qPCR reaction included 10 ng of cDNA, the appropriate forward and reverse primers at the concentrations detailed in Table 1, 12.5 µL Power SYBR Green PCR Master Mix (Applied Biosystems Inc., Foster, CA, USA), and RNase-free water in a final volume of 25 µL. The constitutively expressed genes, *UBC* and *18sRNA*, were used as an internal control to verify the qPCR. To ensure that UBC and 18sRNA were suitable reference genes for this study, we revealed that their expression was stable between the tested tissues and during the oestrous cycle and early pregnancy. Real-time conditions were as follows: Initial denaturation and enzyme activation at 95 °C for 10 min, followed by 40 cycles of denaturation at 95 °C for 15 s, annealing at 60 °C for 1 min and elongation at 70 °C for 1 min. Negative controls were performed by substitution of cDNA with water. All samples were in duplicate. The specificity of amplification was tested at the end of the qPCR by melting curve analysis. Levels of gene expression were calculated using the ΔΔCt method and normalised using the geometrical means of reference gene expression levels: *UBC* and *18sRNA*. The Ct values for all non-template controls were under the detection threshold.

### 4.4. Western Blotting

Tissue preparation and lysis, as well as Western blotting analysis was performed essentially as described by Smolinska et al. [39] with a few modifications. Briefly, equal amounts of porcine MBH, POA and SME protein lysates (30 µg of total proteins) were resolved by SDS-PAGE (12.5% gel) for separating CMKLR1, GPR1, CCRL2 and actin, and then transferred to PVDF membranes and blocked for 1 h at room temperature in Tris-buffered saline Tween-20 (TBST) containing 5% skimmed milk powder. After blocking, PVDF membranes were incubated overnight with rabbit polyclonal CMKLR1 (1 µg/uL, ab230442, Abcam, Cambridge, UK), CCRL2 (3.33 µg/uL, ab85224, Abcam, Cambridge, UK), actin (6.58 µg/uL, A2066, Sigma-Aldrich, St. Louis, MO, USA) antibodies or mouse polyclonal GPR1 (2.0 µg/uL, ab169331, Abcam, UK) in TBST. Actin immunoblots were used as an internal control for equal loading and to quantify porcine CMKLR1, GPR1 and CCRL2 proteins. In order to examine immunoreactive bands, PVDF membranes were incubated with goat anti-rabbit antibodies for CMKLR1, CCRL2 and actin (0.25 µg/uL, sc-2054, Santa Cruz, Santa Cruz, CA, USA) or goat anti-mouse antibodies for GPR1 (0.5 µg/uL, 115-035-003, Jackson ImmunoResearch Laboratories Inc., West Grove, PA, USA) conjugated with horse radish peroxidase (HRP) diluted in TBST containing 5% skimmed milk. PVDF membranes were incubated with Immobilon Western Chemiluminescent HRP Substrate (Merck Millipore, Burlington, MA, USA) and visualised using G: Box EF Gel Documentation System (Syngene, Cambridge, UK) with GeneSnap software. The same protocol was performed in relation to the adipose tissue used as the positive controls. The quality of the experiment was confirmed using reference protein, actin, the expression of which did not vary across the oestrous cycle and pregnancy or between the studied tissues. The results of Western blotting analyses were quantified by densitometric scanning of immunoblots with Image Studio^TM^ Lite version 5.2 software (LI-COR, Lincoln, NE, USA). Data were expressed as the ratio of CHEM receptors proteins relative to actin proteins in arbitrary optical density units.

### 4.5. Enzyme-Linked Immune-Sorbent Assay (ELISA) of CHEM

The concentrations of CHEM protein in plasma were determined using a commercial ELISA kit (FineTest, Wuhan Fine Biotech Co., Ltd., Wuhan, China) according to the manufacturer’s protocol. The range of standard curve was 0.156–10 ng/mL. The sensitivity of the assay was defined as the least protein concentration that could be differentiated from zero samples, which was <0.1 ng/mL. According to the manufacturer, no significant cross-reactivity or interference between CHEM and homologous proteins assayed has been observed. Species cross-reactivity has not been specifically determined. Absorbance values were measured at 450 nm using an Infinite M200 Pro reader with Tecan i-control software (Tecan, Männedorf, Switzerland). The data were linearised by plotting the log of CHEM concentrations versus the log of the optical density and the best fit line was determined by regression analysis. Intra-assay coefficient of variation of the ELISA assay for CHEM was 3.89%.

### 4.6. Statistical Analysis

Data are presented as means ± S.E.M. from five different observations. Differences between groups were analysed by one-way ANOVA followed by Tukey’s honest significant difference *post-hoc* test. Statistical analyses were performed using Statistica Software (StatSoft Inc., Tulsa, OH, USA). Values for *p* < 0.05 were considered statistically significant.

## 5. Conclusions

The presented data indicated, for the first time, the presence of CHEM and its receptors in the hypothalamic structures responsible for GnRH production and secretion, which suggest that the adipokine exerts autocrine/paracrine effects on GnRH synthesis and/or secretion. Moreover, the observed changes in CHEM system expression levels may be dependent on the influence of ovarian steroids and other hormones controlling reproductive processes. Our present findings expand our knowledge of the potential role of CHEM as a key neuromodulator of reproductive functions.

## Figures and Tables

**Table 1:** Characteristics of primers used in the study.

Gene	Primers Sequences	Accession Number	Amplicon Length, bp	Primer, nM	Reference
***RARRES2***	F: 5′-TGGAGGAGTTCCACAAGCAC-3′	EU660865	154	500	[The present study]
	R: 5′-GCTTTCTTCCAGTCCCTCTTC-3′			500	
***CCRL2***	F: 5′-GAGCAGCAGCTACTTACTTCC-3′	NM_001001617.1	196	200	[The present study]
	R: 5′-CTGCCCACTGACCGAGTTC-3′			200	
***CMKLR1***	F: 5′-GGACTACCACTGGGTGTTCG-3′	EU660866	174	200	[The present study]
	R: 5′-GCCATGTAAGCCAGTCGGA-3′			200	
***GPR1***	F: 5′-ACCGACTTGGAGGAGAAAGC-3′	FJ234899.1	159	200	[The present study]
	R: 5′-ATTGAGGAACCAGAGCGTGG-3′			200	
***UBC***	F: 5′-GGAGGAATCTACTGGGGCGG-3′	XM_003483411.3	103	400	[37]
	R: 5′-CAGAAGAAACGCAGGCAAACT-3′			400	
***18sRNA***	F: 5′-TCCAATGGATCCTCGCGGAA-3′	AY265350.1	149	400	[38]
	R: 5′-GGCTACCACATCCAAGGAAG-3′			400	

*RARRES2*: chemerin; *CCRL2*: C-C motif chemokine receptor like 2; *CMLKR1*: chemokine-like receptor 1; *GPR1*: G protein-coupled receptor 1; *UBC*: Ubiquitin C; *18sRNA*: 18S ribosomal RNA; F: forward; R: reverse.
